# Dandy-Walker malformation and psychotic disorder. Review in accordance with a clinical case

**DOI:** 10.1192/j.eurpsy.2024.380

**Published:** 2024-08-27

**Authors:** A. Calle González, D. Batet Sánchez, Á. Martínez Hernanz, I. Roque González

**Affiliations:** Psychiatry, Hospital Universitario de Getafe, Getafe, Spain

## Abstract

**Introduction:**

A clinical case is presented of an 18-year-old woman diagnosed with Dandy-Walker malformation, who is admitted to an Acute Inpatient Psychiatry Unit due to atypical psychotic symptoms, pseudology and aggressive behaviour. After several medication trials, there is a partial response observed with low doses of clozapine, consolidating the improvement afterwards, being referred to an open-door community mental health center because of poor family and social network.

**Objectives:**

Review clinical information about Dandy-Walker malformation and the development of psychiatric disorders, specifically psychotic symptoms, pointing out the peculiarities regarding clinical presentation and treatment management.

**Methods:**

Search in the medical database PUBMED, MEDSCAPE and UPTODATE. Keywords: “Dandy-Walker Syndrome”,” Psychotic Disorders”.

**Results:**

The Dandy-Walker syndrome consists on a cystic dilatation of the fourth ventricle, an abnormally high tentorium and the agenesis of the cerebellar vermis. Cerebellar structures are involved in cognitive, emotional and behavioural processes. This syndrome is related to the development of psychotic and affective disorders, as well as obsessive-compulsive disorder. The clinical presentation is usually atypical, being characterised by an early onset, a family history of psychosis and a high prevalence of cognitive deficit and borderline intelligence. There are no specific drugs recommended for the treatment of these patients, which present a high rate of refractoriness to antipsychotic treatments, together with a greater sensitivity to its side effects. Depending on the clinical presentation it is advisable to focus on the most relevant symptoms to be treated and potential side effects in order to reduce polypharmacy.

**Image:**

**Figure:**
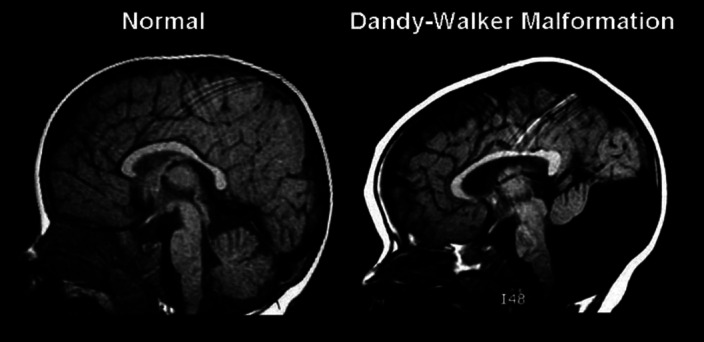

**Conclusions:**

- The Dandy-Walker syndrome is related to a higher risk of psychiatric disorders
- Clinical presentation is usually atypical and in early stages
- There is a high rate of refractoriness and greater sensitivity to treatments
- A specific pharmacological treatment is not recommended and it is recommended to avoid polypharmacy

**Disclosure of Interest:**

None Declared

